# Students' Perceptions of Learning Life Skills Through the Teaching Personal and Social Responsibility Model: An Exploratory Study

**DOI:** 10.3389/fspor.2022.898738

**Published:** 2022-05-27

**Authors:** Jennifer M. Jacobs, Paul M. Wright, K. Andrew R. Richards

**Affiliations:** ^1^Department of Kinesiology and Physical Education, Northern Illinois University, DeKalb, IL, United States; ^2^Department of Kinesiology and Community Health, University of Illinois at Urbana–Champaign, Champaign, IL, United States

**Keywords:** positive youth development (PYD), models-based instruction, values-based education, social and emotional learning (SEL), professional development (PD)

## Abstract

**Purpose:**

Physical education (PE) lags behind community-based sport and physical activity programs in the integration of positive youth development (PYD) principles and practices such as teaching transferable life skills. However, research and educational policy indicates this can and should be part of the PE curriculum. Therefore, there is a significant need to explore students' perceptions and experiences about learning life skills within the PE context. In the current study, an intervention based in a wellestablished PYD approach called Teaching Personal and Social Responsibility (TPSR), was delivered to assess these issues.

**Methods:**

The current study was conducted in the mid-western U.S. Participants were 122 adolescent students (m = 60, f = 62; *M* = 12.48 years, SD = 0.97 years) in intervention and control classes. For the intervention, a PE teacher received training on the TPSR approach to promote life skills, while the control teacher received no training and participated in usual practices. Pre- and post-surveys were distributed that examined student perceptions about learning life skills, and supplemental systematic observations were recorded to capture the intervention teacher's fidelity to the TPSR model.

**Results:**

Results indicated that the intervention group students' perceptions of in-class experiences with life skills such as problem solving, emotional regulation, effort, goal setting, identity experiences, time management, and promoting social norms were enhanced overtime, compared to the control group.

**Conclusion:**

PE is in a unique position to promote PYD in the school curriculum by teaching of life skills. In this case, participants in the intervention group demonstrated learning personally and socially responsible behaviors across the course of 15 PE lessons. Future research should examine if changed in-class perceptions about life skills can foster use of these skills outside of the PE setting.

## Introduction

While adolescents form their identities, enjoy increased independence, and establish lifestyle habits, a host of personal and social skills are crucial to helping them make healthy and responsible choices (Catalano et al., 2002). For example, research has shown those with higher levels of social and emotional competency tend to do better academically and avoid trouble in school (Taylor et al., 2017). Given the same intellectual ability, a student who exercises persistence and self-management skills is more likely to achieve good grades because they complete homework and study for tests. A student who has a greater sense of emotional awareness and impulse control is less likely to escalate a verbal conflict to a physical one. However, helping children and youth to develop such skills and apply them when called upon is no small feat, especially when these skills are inconsistent with norms and expectations in their social environments (Jacobs and Wright, 2021).

Positive youth development (PYD) is a strength-based approach to support youth, especially those from marginalized communities, by nurturing values, attitudes, and behaviors that can help them to navigate challenges and thrive in their various roles and responsibilities (Catalano et al., 2002). Because PYD strives to provide engaging, social, and empowering experiences, sport and other physical activity programs are ideal settings for doing this work (Hellison et al., 2000; Holt, 2016). A common focus of physical activity based PYD programs is teaching transferable *life skills*, i.e., skills that can be demonstrated, learned, and discussed in one context for their eventual use in other environments (Gould and Carson, 2008; Jacobs and Wright, 2018). Examples of life skills that are readily developed in physical activity and sport include self-control, communication, teamwork, leadership, and goal-setting (Gould and Carson, 2008). The broader educational literature indicates programs that effectively promote transferable personal, social, and emotional skills like these can positively impact youth in terms of their mental health, academic performance, and overall wellbeing (Taylor et al., 2017).

An emphasis on PYD and life skill education in sport and physical activity has taken root in community settings such as after-school programs and summer camps (Hellison et al., 2000; Gould and Carson, 2008; Gordon et al., 2016; Holt, 2016; Jacobs and Wright, 2018, 2021). However, the PYD framework is not often applied in school-based PE despite its apparent relevance (Wright and Li, 2009) and a strong body of literature focused on students' development of personal and social skills within PE (Hemphill et al., 2015; Pozo et al., 2016; Opstoel et al., 2020) as well as their affective development (Teraoka et al., 2020). These aspects of PE not only align with PYD outcomes, but also with PE content standards in many nations such as the U.S., New Zealand and Scotland (Richards and Gordon, 2017; Wright and Irwin, 2018; Wright et al., 2021).

One possible explanation for the limited use of the PYD framework in PE is the fact that PYD has been largely developed and applied in community settings rather than the school curriculum (Hellison et al., 2000; Catalano et al., 2002; Holt, 2016). Another possible explanation is the pressure PE teachers are under to address numerous curricular standards and expectations, often with large numbers of students and limited time (Hellison, 2011). Additionally, while PE teachers often demonstrate strong values related to developing the affective domain, rarely are they trained, evaluated, or held accountable for promoting life skills within their teaching (Gordon, 2010; Richards and Gordon, 2017; Wright et al., 2021).

Although the terminology of PYD has not been widely applied in PE, there has been growing interest in a highly related framework, i.e., social and emotional learning (SEL: Jacobs and Wright, 2014; Wright and Irwin, 2018; Dyson et al., 2021; Wright and Richards, 2022). The field of SEL, a robust approach to youth development (Taylor et al., 2017), promotes a set of intra- and interpersonal behaviors and competencies, like life skills, that are increasingly called for in PE and across other subject areas (Jacobs and Wright, 2014; Wright and Richards, 2022). With this focus on SEL in the school curriculum, PE teachers have an opportunity to align their promotion of personal and social skills, affective development, etc. with a broader educational movement as well as their own content standards (Jacobs and Wright, 2014; Wright and Richards, 2022). For example, in the U.S. context, national standards that guide PE practices (SHAPE America, 2019) encourage teachers to foster affective development as well as SEL outcomes (Dyson et al., 2021; Wright and Richards, 2022). Concepts such as personal and social responsibility, cited directly in the U.S. national standards, can be reflected in a number of personal and social skills such as showing respect, controlling one's temper, helping others, cooperating, etc. Despite these strong alignments, researchers (Gordon, 2010; Hemphill et al., 2015; Richards and Gordon, 2017; Wright and Irwin, 2018; Wright et al., 2021) have noted that it is difficult for physical educators to conceptualize how one can effectively implement personal and social skills into PE without losing a focus on physical skill development.

One way to support progress in this area is to study life skills directly to make an empirical case for PE experiences having the capacity to foster PYD (and hence SEL) outcomes. Valid and reliable methods that examine which life skills students report learning through PE could foster this solution (Pozo et al., 2016; Teraoka et al., 2020; Dyson et al., 2021). In this study we sought to explore how students perceived learning life skills in a PE class taught using a PYD oriented life skill education approach called the Teaching Personal and Social Responsibility model (TPSR; Hellison, 2011) compared to students in a class where life skills were not explicitly emphasized.

### The Teaching Personal and Social Responsibility Model

Sport and physical activity have been promoted as effective vehicles for teaching life skills due to the natural occurrence of opportunities to learn lessons that pertain to life (Hellison et al., 2000). For example, sport inherently introduces situations related to winning and losing, exhibiting focus and effort, and interacting with peers from different backgrounds, which present opportunities to connect learning in PE to outside the gymnasium (Wright and Richards, 2022). One welldeveloped approach for teaching life skills in PE is the TPSR model (Hellison, 1985, 2011). TPSR is a student-centered approach that gradually empowers and challenges students to take responsibility for their own learning and for the wellbeing of others. TPSR is recognized in PE as a best practice as a best practice for promoting affective development as well as teaching personal, social, and emotional skills (Metzler, 2011; Pozo et al., 2016; Opstoel et al., 2020; Teraoka et al., 2020; Dyson et al., 2021).

After several decades of refinement, Hellison (2011) framed the TPSR model around five major responsibility goals. These goals, often organized into a loose progression of levels, are presented below with common examples of associated skills and behaviors.

1. ***Respecting the rights and feelings of others***, e.g., self-control, inclusion, peaceful conflict resolution.
2. ***Self-motivation***, e.g., effort, persistence, trying new things.
3. ***Self-direction***, e.g., making decisions, working independently, setting goals.
4. ***Caring***, e.g., helping or encouraging others, leadership, considering the group's welfare.
5. ***Transfer***, e.g., applying ideas, values, and responsible behaviors from the program in other settings and situations such as home or the classroom.

PE lessons informed by the TPSR model can be designed to incorporate specific life skills such as respect, self-control, and leadership so that students may practice these behaviors in a supervised setting while being encouraged to transfer them to other life areas (Wright and Burton, 2008; Pozo et al., 2016). For example, during a soccer lesson, a PE teacher might describe effort as trying hard when motivation falters. The teacher could extend the idea by asking students to rate their effort during the lesson and discuss how putting forth effort in soccer can relate to exam preparation or making new friends. Most TPSR lessons follow a common lesson outline (Hellison, 2011) that includes time for relationship-building, an awareness talk, life skill-based sport instruction, and reflection time with a specific emphasis on how the practiced life skills can be applied in other environments.

### Pedagogical Model Fidelity

While most TPSR lessons follow a common outline, one reason the model has been widely implemented in PE is because it provides a flexible and comprehensive way to integrate life skills into sport (Hellison, 2011). According to Dyson and Casey (2012), it is common for teachers to modify their models-based instruction to suit their preferences. Gordon (2010) adds that variation in teaching may be a result of teachers having a strong understanding of the foundations of the model in a way that enables them to address student needs while still adhering to model goals. As is crucial in any models-based instruction, best practices include understanding the needs of the students, connecting to school-wide initiatives, and seeking ongoing professional support to ensure implementation fidelity (Metzler, 2011). Moreover, Hastie and Casey (2014) suggest that investigations into any models-based practice in PE should address fidelity by providing a rich description of the curricular content, a customized method to validate model implementation, as well as an explanation of the teachers' and students' prior experience with the model.

Flexibility in model implementation has always been advocated in the TPSR literature (Hellison, 2011), but there is a concurrent need to maintain adherence to general model principles to avoid a *toxic mutation* (Gordon et al., 2016) whereby the model retains its identity in name only. Toward this end, TPSR research has demonstrated a relationship between implementation fidelity (i.e., the degree to which teacher practice aligns with theoretical underpinnings, inherent philosophy, and applied components of the model) and positive student outcomes. Fidelity is fostered through a process of providing teachers with practical and effective instructional strategies that reflect teaching models (Pascual et al., 2011). Evidence suggests that teachers are more likely to implement a pedagogical model with fidelity, for example, when they are provided protracted opportunities to practice, reflect upon, and receive feedback on implementation in an environment that includes ongoing support (Escartí et al., 2010).

### Continuing Professional Development

Given that the TPSR model includes flexible guidelines for implementation, one commonly observed teacher barrier is confusion over how to employ TPSR strategies in PE (Richards and Gordon, 2017). Accordingly, continuous professional development (CPD) programs have been emphasized as a critical step in helping PE teachers incorporate TPSR into their practice (Hemphill et al., 2015). Wellstructured CPD programs should include key elements of collaboration, teacher ownership, practicality, ongoing feedback, and ample time for the teacher to learn and implement new practices (Hemphill et al., 2015). Systematic observation tools have been advocated as one method for supporting responsibility-based professional development in PE (Hemphill et al., 2015). In line with best practices from CPD, The Tool for Assessing Responsibility-Based Education (TARE) 2.0 (Escartí et al., 2015), can be used to provide teachers feedback on their implementation of concrete instructional strategies that are consistent with TPSR. As such, Hemphill et al. (2015) illustrated how the TARE 2.0 could be used to promote active participation of teachers throughout the learning process, foster reflection on and awareness of responsibility-based strategies, and thus, increase the overall likelihood they will implement these strategies effectively. Similar results have been reported in action research projects conducted by PE teachers using the strategies contained in the TARE 2.0 to guide their improvement (Coulson et al., 2012; Gray et al., 2019).

Despite support for using TPSR as a method for promoting positive outcomes in PE (Metzler, 2011), one limit of TPSR research is that there is not a sufficient amount of studies examining teachers' ability to learn the model that connect with student outcomes. Additionally, there are few, if any, studies that use quasi-experimental designs to understand how interventions can improve teachers' use of TPSR in relation to a control group. Therefore, the current study's design was inspired by literature that calls for research to (a) describe how PE experiences influence students' life skill-learning, and (b) use a validated observation tool to examine if implementing fundamental TPSR teaching strategies in an intentional way mediates this process. Exploring this line of inquiry could uncover how professional development programs that help practicing teachers learn TPSR can result in measurable, positive developmental outcomes for students. Specifically, the purpose of this study was to examine how students in a TPSR-based PE unit interpreted their experience with respect to learning life skills as compared to students taught using traditional PE practices. The following research questions guided the inquiry: 1) How does a PE teacher's instruction using the TPSR model impact model fidelity as a result of professional development training? 2) How does instruction through the TPSR model influence students' learning of life skills in PE when compared to traditional instructional practices?

## Methods

### Participants and Setting

Participants in this study were 122 students (m = 60, f = 62) from a middle school located in a small city in the U.S Midwest. At the time of the study, the school had 644 sixth-, seventh-, and eighth-grade students and 48% percent of the students were identified as low income based on receiving free or reduced lunch. Eligibility criteria included enrollment in one of three classes taught by either the control teacher or intervention teacher. Across these six classes (i.e., two per each grade level), a total of 173 students were eligible to participate in the study. The final sample consisted of 122 students (62 male, 60 female) from sixth (*n* = 32; *M* = 11.47 years, SD = 0.51 years), seventh (*n* = 54; *M* = 12.30 years, SD = 0.54 years), and eighth (*n* = 36; *M* = 13.64 years, SD = 0.49 years) grade classes who returned assent forms and completed both pre- and post-surveys. The majority of the participants were Caucasian (44%) with the remaining identifying as African American (20%), Hispanic (17%), two or more races (17%), or Asian (1.5%). Both teachers taught volleyball units during the study and had between 25 and 30 students per class.

The intervention teacher, assigned the pseudonym “Olivia,” was a Caucasian female and had taught PE for 19 years at the middle-school level. Olivia had been a part of a professional development partnership with the local university for several years, which included co-teaching classes with university faculty using models-based instruction (e.g., sport education, adventure education). Olivia was recruited for participation in the current study based on the prior knowledge that her ongoing professional development training gave her experience with models-based teaching. However, she did not have prior experience implementing the TPSR model.

The control teacher, who was assigned the pseudonym “Ben,” was a Caucasian male who had been teaching PE for 10 years at the middle-school level. Ben was not part of the university's professional development group and therefore received no formal training on models-based instruction in PE. However, the university faculty had also known Ben for several years through the broader partnership with the school. Based on informal observations, conversations with Ben, and anecdotal comments spanning several years, the faculty had the impression that his teaching approach focused on direct instruction, followed by game play, with little explicit emphasis on the affective domain in a way that is advocated through TPSR. Baseline data shared later will provide evidence supporting this assertion. Altogether, similar to Wright and Irwin (2018), teachers were recruited based on pre-existing knowledge of their general approach and dispositions and later, systematic data collection was used to document their styles empirically and with more precision.

### Research Design

The current study employed a quasi-experimental research design with intervention and control groups tested before and after the intervention period (Cook, 2015). Both Olivia's and Ben's students received a baseline survey assessing the extent to which they believed that their teacher provided responsibility-based experiences in PE. Next, baseline observational data using the TARE 2.0 (Escartí et al., 2015) were collected in five of Olivia's and Ben's class periods in order to describe their standard use of responsibility-based teaching strategies. After the initial observation period for both teachers, job-embedded professional development was provided to the intervention teacher to educate her on specific TPSR teaching strategies. She then implemented this style of teaching as an intervention for 4 weeks, spanning the volleyball unit, which amounted to 15 total class sessions. Both teachers were observed using systematic observation, across the same time period, which comprised their volleyball units. Students of both teachers received an identical post-survey instrument at the end of the intervention.

### Overview of the Intervention

Olivia received training from the first two authors of the study, who have 25 years of combined experience with TPSR including designing and teaching youth programs, training teachers and coaches in the model, and evaluating TPSR programs. The training included strategies for incorporating responsibility-based teaching informed by the TPSR model into PE. Specifically, four empowerment-based teaching strategies (i.e., giving students voices, promoting student leadership, fostering student reflection through discussion, and teaching for transfer) were emphasized. A customized training manual, which included volleyball activities and potential life skill discussion prompts was devised by the first author for Olivia's use. In total, the intervention teacher training spanned two initial sessions of two hours each. Additionally, because the first author served as the observer for daily class lessons, informal daily debriefing sessions (i.e., ranging from 5 to 15 mins) were conducted before and after classes as a manner for providing formative feedback. These approaches to professional development (e.g., teaching model fidelity, providing sample lesson plans, having briefing and debriefing sessions) align with prior research on introducing model-based instruction through on-site professional development with physical educators (Sinelnikov, 2009; Goodyear, 2017).

In line with the TPSR model (Hellison, 2011), Olivia incorporated at least one new responsibility-based activity that focused on a life skill (e.g., effort, self-control) into each of the 15 intervention classes. Examples of tactics integrated include peer coaching during serving drills, group discussions on class effort, and exit slips regarding self-control. Previous research has demonstrated students being exposed to responsibility-based teaching with a similar frequency as sufficient to influence their perceptions of life skill instruction (Wright and Burton, 2008). Ben participated in his usual teaching practices focused on building sport competency in volleyball (e.g., passing, serving) structured primarily through skill stations and scrimmages.

### Data Collection

Prior to data collection, the lead researcher's Institutional Review Board granted approval for the study, and all necessary approvals were gained from the school district, teachers, parents, and students. Two identical surveys were given that contained demographic questions and a 40-item version of the Youth Experience Survey 2.0 (YES 2.0; Hansen and Larson, 2005), and classes for both teachers were coded using the TARE 2.0 across multiple lessons before and during the intervention (Escartí et al., 2015).

#### Students' Perceptions of Life Skills Learning

The YES 2.0 (Hansen and Larson, 2005) evaluates adolescents' perceptions of in-program, positive developmental experiences, both personal and interpersonal, that promote learning life skills such as leadership, responsibility, and effort (Hansen and Larson, 2005). Selected scales totaling 40 items (i.e., identity exploration [three items], identity reflection [three items], goal setting [three items], effort [three items], problem solving [three items], time management [three items], emotional regulation [four items], diverse peer relationships [four items], prosocial norms [four items], group processing skills [five items], feedback [two items], and leadership/responsibility [three items]) were taken from the original 70-item survey based on relevance to the PE setting and alignment with TPSR. For each item, participants rated whether they had a given experience in PE both prior to and directly following the intervention period. Responses were set to a 4-point, Likert-type scale ranging from “yes, definitely” (1) to “not at all” (4). Example items included “PE class got me thinking about who I am” (identity exploration), “this PE class got me thinking about who I am” (identity reflection), “I set goals for myself in PE class” (goal setting), “in PE class I learned to push myself” (effort), “used my imagination to solve a problem” (problem solving), “learned about controlling my temper” (emotional regulation), “made friends with someone of the opposite gender” (diverse peer relationships), “learned about helping others” (prosocial norms), “became better at sharing responsibility” (group process skills), “I became better at giving feedback” (feedback), and “learned about the challenges of being a leader” (leadership and responsibility). Internal consistency reliability for the YES 2.0 has been demonstrated in previous research (Strachan et al., 2009; Cronbach's α = 0.77 to 0.94).

#### Implementation of Responsibility-Based Teaching Strategies

Hastie and Casey (2014) recommend incorporating customized fidelity instruments (e.g., fidelity checklists) to assess the implementation of models-based practice in PE. In the current study, this was accomplished with The TARE 2.0 (Escartí et al., 2015), which was used as a live coding systematic observation instrument to assess the integration of life skills instruction in accordance with the principles of TPSR. The development of the TARE 2.0 was informed by scholars' immersion in TPSR practice and research and resulted in the development of nine essential teaching strategies and nine student behaviors presumed to result from high-quality responsibility-based teaching (see Tables 1, 2 for complete list of behaviors). It uses direct observation and time sampling of 3-min intervals to assess teacher and student behaviors during physical activity instruction. For each 3-min interval, the behaviors are rated from zero (absent) to 4 (very strong). In the current study, the TARE 2.0 was utilized by the first author to collect baseline and post-intervention data from both Olivia and Ben's classes. The first author had undergone approximately 20 hours of training and supervised practice using the TARE 2.0 by the second author, one of the instrument's developers. During the training phase, the two consistently achieved more than 80% inter-rater agreement.

**Table 1:** Pre- and post-TARE 2.0 ratings of teacher and student behaviors for the intervention teacher.

**Behavior**	**Pre-score *M* (*SD*)**	**Post-score** ***M*** **(*SD*)**	***T*** **-test results**		
			***t*** **-statistic**	***p*** **-value**	**Cohen's *d***
**Teacher behaviors**					
Modeling respect	3.70 (0.46)	3.83 (0.4)	1.87	0.063	0.30
Setting expectations	3.72 (0.53)	3.71 (0.54)	0.11	0.916	0.02
Opportunities for success^*^	3.13 (0.62)	3.59 (0.57)	4.95	< 0.001	0.79
Fostering social interaction^*^	3.19 (0.59)	3.56 (0.65)	3.73	< 0.001	0.59
Assigning tasks	3.43 (0.69)	3.53 (0.56)	1.13	0.260	0.18
Leadership^*^	0.69 (0.97)	3.02 (0.80)	17.39	< 0.001	2.77
Giving choices and voices^*^	0.94 (1.20)	3.04 (1.13)	11.45	< 0.001	1.83
Role in assessment^*^	0	1.31 (1.53)	6.30	< 0.001	1.00
Transfer^*^	0	0.71 (1.29)	4.05	< 0.001	0.65
**Student behaviors**					
Participation	3.89 (0.32)	3.82 (0.43)	1.07	0.288	0.17
Engagement	3.74 (0.48)	3.65 (0.53)	1.06	0.290	0.17
Showing respect	3.41 (0.71)	3.40 (0.74)	0.06	0.949	0.01
Cooperating with peers	3.31 (0.67)	3.40 (0.70)	0.77	0.441	0.12
Encouraging others^*^	2.65 (0.76)	3.19 (0.69)	4.84	< 0.001	0.77
Helping others^*^	2.17 (0.97)	2.71 (0.98)	3.49	0.001	0.56
Leading^*^	0.81 (1.07)	2.76 (0.84)	13.54	< 0.001	2.16
Expressing voice^*^	1.00 (1.20)	3.14 (0.91)	13.55	< 0.001	2.16
Asking for help^*^	0.46 (0.79)	1.22 (1.03)	4.90	< 0.001	0.78

*All TARE 2.0 categories were measured on a five-point rating scale with 0 indicating that the behavior was absent and 4 indicating that it was very strong, n = 54 observation segments pre-intervention, n = 150 observation segments post observation,*

* *p < 0.05*.

**Table 2:** Pre- and post-TARE 2.0 ratings of teacher and student behaviors for the control teacher.

**Behavior**	**Pre-score *M* (*SD*)**	**Post-score *M* (*SD*)**	***T*** **-test results**		
			***t*** **-statistic**	***p*** **-value**	**Cohen's *d***
**Teacher behaviors**					
Modeling respect^*^	2.5 (0.66)	2.78 (0.59)	2.84	0.005	0.45
Setting expectations^*^	2.4 (0.88)	3.41 (0.84)	7.49	< 0.001	1.19
Opportunities for success^*^	1.79 (0.76)	2.51 (0.79)	5.82	< 0.001	0.93
Fostering social interaction^*^	1.59 (0.97)	2.82 (0.81)	8.91	< 0.001	1.42
Assigning tasks^*^	2.04 (0.2)	2.76 (0.83)	5.45	< 0.001	0.87
Leadership^*^	0.19 (0.58)	1.00 (1.22)	5.09	< 0.001	0.81
Giving choices and voices^*^	0.15 (0.47)	0.63 (1.05)	3.57	< 0.001	0.57
Role in assessment^a^	0	0	–	–	–
Transfer^a^	0	0	–	–	–
**Student behaviors**					
Participation	3.89 (0.32)	3.82 (0.43)	1.74	0.083	0.28
Engagement^*^	3.74 (0.48)	3.65 (0.53)	2.12	0.036	0.34
Showing respect^*^	3.41 (0.71)	3.40 (0.74)	2.45	0.015	0.39
Cooperating with peers^*^	3.31 (0.67)	3.40 (0.70)	3.21	0.002	0.51
Encouraging others^*^	2.65 (0.76)	3.19 (0.69)	3.93	< 0.001	0.62
Helping others	2.17 (0.97)	2.71 (0.98)	0.53	0.595	0.08
Leading^*^	0.81 (1.07)	2.76 (0.84)	4.44	< 0.001	0.71
Expressing voice^*^	1.00 (1.20)	3.14 (0.91)	3.58	< 0.001	0.57
Asking for help	0.46 (0.79)	1.22 (1.03)	1.54	0.125	0.24

*All TARE 2.0 categories were measured on a five-point rating scale with 0 indicating that the behavior was absent and 4 indicating that it was very strong, n = 68 observation segments pre-intervention, n = 98 observation segments post observation,*

* *p < 0.05,*

a *tests not performed because there were no observed instances of the behavior*.

### Data Analysis

Steps were taken to ensure that the final sample included data that were accurate and reliable (Tabachnick and Fidell, 2013). Specifically, before running the primary data analyses, standard diagnostic tests to check for normality, linearity, and homogeneity of variance were conducted. Additionally, internal consistency assessments for all scales and subscales were conducted using Cronbach (1951) coefficient alpha. Items were averaged into constructs and both descriptive statistics and bivariate correlations were calculated.

The primary analyses included independent-samples *t*-tests to examine changes in the TARE 2.0 student and teacher behaviors before and after the intervention as well as 2 × 2 (Time × Condition) mixed ANOVAs for each subscale of the YES 2.0. The 2 × 2 (Time × Condition) mixed ANOVAs examined the influence of the main effects (i.e., time and condition) and the interaction effect on students' perceptions of life skills learning as measured by the YES 2.0. Partial-η^2^ was used as a measure of effect size in the mixed ANOVA models. A partial-η^2^ value between 0.01 and 0.06 is associated with a small effect, between 0.06 and 0.14 with a medium effect, and > 0.14 with a large effect (Warner, 2012). Significant interaction effects were followed up with tests for simple effects using paired-samples *t* tests. Cohen's d was used as a measure of effect size for *t-*tests where a value between 0.2 and 0.5 is associated with a small effect, between 0.5 and 0.8 a medium effect and > 0.8 with a large effect (Cohen, 1992). All statistical analyses were conducted using IBM SPSS 23.0.

## Results

In order to document intervention fidelity, the TARE 2.0 results are reported in Tables 1, 2. In general, results support the notion that (1) the intervention was carried out with fidelity, and (2) based on survey data, participants in the intervention group were exposed to more responsibility-based pedagogy after the intervention.

### Implementation of Responsibility-Based Teaching Strategies

The TARE 2.0 (Escartí et al., 2015) was used to examine the extent to which teacher and student behaviors associated with responsibility-based teaching were used in both Olivia's (intervention teacher) and Ben's (control teacher) classes at pre- and post-intervention. Olivia's ratings were generally high across teacher and student behaviors at the pre-test and most scores increased after the intervention (see Table 1). The following teaching strategies increased from pre- to post-intervention: opportunities for success, fostering social interactions, leadership, giving choices and voices, role in assessment, and transfer. Olivia's ratings for modeling respect, setting expectations, and assigning tasks did not change pre- to post-intervention, although they were already quite high to start. The ratings of student behaviors after the intervention showed significant increases with encouraging others, helping others, leadership, expressing voice, and asking for help. Student behaviors associated with participation, engagement, showing respect, and cooperating with peers did not change significantly.

Ben's rating on some of the TARE 2.0 (Escartí et al., 2015) teaching and student behaviors also increased from pre- to post-observation, despite the fact that he did not participate in the intervention. His scores (see Table 2) did, however, start off rather low and therefore had more room for improvement. In terms of the teaching behaviors, modeling respect, setting expectations, opportunities for success, fostering social interactions, assigning tasks, leading, and giving choices and voices all increased. Ben's scores related to providing a role in assessment and promoting transfer were rated as 0 (not present), so these tests were not run. The ratings of student behaviors after the intervention showed significant increases with engagement, showing respect, cooperating with peers, encouraging others, leading, and expressing voice. No differences were observed relative to participation, helping others, and asking for help.

### Perceptions of Life Skill Learning

Next, 2 × 2 (Time × Condition) Mixed ANOVAs were conducted to examine the changes in the YES 2.0 subscales while considering the condition (i.e., intervention or control) as a moderating variable. Table 3 overviews the results of these tests for the YES 2.0 subscales, including descriptive statistics and internal consistency reliability estimates, and significant effects are discussed in the paragraphs that follow.

**Table 3:** ANOVA table for the subscales of the YES 2.0 by teacher and time.

**Subscale time**	**Teacher type**				**ANOVA statistics**		
	**Intervention teacher *M (SD)***	**Control teacher *M (SD)***	**α**	**Factor**	***F***	***P***	**Partial-η^2^**
Identity exps				Time	3.70	0.082	0.025
Pre	2.51 (0.78)	2.46 (0.74)	0.71	Teacher	2.85	0.094	0.023
Post	2.79 (0.79)	2.42 (81)	0.81	Interaction^*^	4.65	0.033	0.037
Identity refl				Time	3.00	0.086	0.024
Pre	2.00 (0.94)	2.27 (1.00)	0.87	Teacher	3.36	0.069	0.027
Post	1.85 (0.86)	1.86 (0.92)	0.92	Interaction	2.61	0.109	0.021
Goal setting				Time	0.24	0.626	0.002
Pre	2.44 (0.95)	2.20 (0.97)	0.88	Teacher^**^	7.90	0.006	0.062
Post	2.66 (0.91)	2.06 (0.88)	0.92	Interaction^*^	5.34	0.023	0.043
Effort				Time	0.12	0.733	0.001
Pre	2.71 (0.83)	2.68 (0.92)	0.81	Teacher	1.71	0.194	0.014
Post	2.84 (0.90)	2.50 (0.92)	0.87	Interaction^*^	5.20	0.024	0.042
Problem solve				Time	0.012	0.912	0.000
Pre	2.27 (0.92)	2.43 (0.97)	0.87	Teacher	0.479	0.490	0.004
Post	2.54 (0.89)	2.16 (0.86)	0.87	Interaction^**^	14.6	< 0.001	0.108
Time mang				Time	0.45	0.502	0.004
Pre	2.38 (0.97)	2.26 (0.92)	0.87	Teacher^*^	4.87	0.029	0.039
Post	2.64 (0.90)	2.10 (0.90)	0.90	Interaction^**^	7.67	0.006	0.060
Emo regulat				Time	1.61	0.207	0.013
Pre	2.20 (0.92)	2.25 (0.93)	0.87	Teacher	0.952	0.331	0.008
Post	2.48 (0.91)	2.15 (0.84)	0.89	Interaction^*^	6.82	0.010	0.054
Diverpeerrelt				Time	1.00	0.320	0.008
Pre	2.63 (0.82)	2.54 (0.93)	0.86	Teacher	0.72	0.397	0.006
Post	2.60 (0.92)	2.44 (0.89)	0.91	Interaction	0.36	0.551	0.003
Prosocnorm				Time^**^	3.73	0.056	0.030
Pre	2.21 (0.87)	2.18 (1.00)	0.90	Teacher	2.24	0.137	0.018
Post	2.54 (0.91)	2.10 (0.93)	0.89	Interaction^**^	8.71	0.004	0.068
Groupprocc				Time	0.012	0.912	0.000
Pre	2.71 (0.80)	2.43 (0.92)	0.91	Teacher^**^	8.74	0.004	0.835
Post	2.84 (0.86)	2.31 (0.83)	0.93	Interaction	3.24	0.074	0.026
Feedback				Time	0.001	0.970	0.000
Pre	2.54 (0.94)	2.38 (0.91)	0.87	Teacher^*^	4.71	0.032	0.038
Post	2.69 (0.98)	2.22 (0.91)	0.88	Interaction	2.99	0.087	0.024
Leaderresp				Time	0.035	0.852	0.000
Pre	2.56 (0.98)	2.52 (0.99)	0.87	Teacher	1.61	0.207	0.013
Post	2.70 (1.00)	2.35 (0.96)	0.89	Interaction	3.02	0.085	0.025

*Identity Exps, Identity Experiences; Iden Refl, Identity Reflection; Problem Solve, Problem Solving; Time Mang, Time Management; Emo Regulat, Emotional Regulation; Phy Skills, Physical Skills; Diver Peer Relt, Diverse Peer Relations; ProSocNorms, Promoting Social Norms; Group Procc, Group Processing Skills; Leader Resp, Leadership and Responsibility;*

* *p < 0.05,*

** *p < 0.01*.

For *problem solving*, the main effects for time and condition were not significant. However, there was a significant interaction for time × condition. Paired-samples *t*-tests indicated that, for the intervention teacher, there was a significant increase over time on students' perceptions of problem solving *t*_(66)_ = 2.78, *p* = 0.007, *d* = 0.48, but there was a significant decrease over time for the control condition, *t*_(54)_ = −2.69, *p* = 0.010, *d* = 0.52.

Related to *effort*, the main effects for time and condition were not significant. However, there was a significant interaction effect, indicating that students in the intervention class perceived an increase in effort, while students in the control group perceived a decrease. Nevetherless, paired samples *t*-tests returned no significant differences between the teachers.

Related to *goal setting*, the main effect for time was not significant, but the main effect of condition was significant. Additionally, the interaction of time × condition was significant. Paired-samples *t*-tests indicated there was an increase in students' perceptions of identity experience in the intervention group, *t*_(66)_ = 2.07, *p* = 0.043, *d* = 0.36, but no change in the control.

For *emotional regulation*, the main effects for time and condition were not significant, but there was a significant interaction effect. A paired-samples *t*-test indicated that intervention students' perceptions of emotional regulation increased over time, *t*_(66)_ = 2.86, *p* = 0.006, *d* = 0.50, but there was no change for the control group.

Related to *identity experiences*, the main effects for time and condition were not significant. However, there was a significant interaction effect of time × condition. Paired samples *t*-tests indicated a significant increase over time on students' perceptions of identity experiences in the intervention group, *t*_(66)_ = 2.95, *p* = 0.004, *d* = 0.51, but not in the control group.

For *prosocial norms*, the main effect for time and main effect for condition were not significant. However, the interaction of time × condition was significant. Paired sample *t*-tests showed a significant increase over time on students' perceptions of prosocial norms in the intervention group, *t*_(66)_ = 3.62, *p* = 0.001, *d* = 0.63, but no change in the control.

Regarding *time management*, the main effect for time was not significant, but the main effect of condition was significant. Additionally, there was a significant interaction of time × condition. Paired samples *t*-tests indicated that there was a significant increase over time on students' perceptions of identity experiences in the intervention group, *t*_(66)_ = 2.21, *p* = 0.030, *d* = 0.38, but no change in the control group.

Related to *group processing*, the main effect for time and interaction effect for time × condition were not significant. However, the condition effect was significant indicating that students in the intervention group perceived higher group processing regardless of time.

For *feedback*, the main effect for time and interaction effect for time × condition were not significant. However, the condition effect was significant, indicating that students in the intervention group percieved more feedback than in the control group regardless of time.

No significant time, condition, or interaction effects were observed for *identity reflection* and *leadership and responsibility*. This indicates that students did not demonstrate differences in reflecting on their identity or leadership and respect between conditions or over time.

## Discussion

The purpose of this study was to examine how responsibility-based teaching in PE influences students' in-class experiences with learning life skills. Results from the TARE 2.0 indicated that the intervention was implemented with fidelity, which was reflected by an increase in teaching strategies and student behaviors reflective of the TPSR model (Escartí et al., 2015). Relatedly, students in the intervention group reported increases in select life skills as measured by the YES 2.0 as compared to a control group.

Results using the TARE 2.0 generally support the idea that when foundational teaching practices are implemented regularly (e.g., modeling respect, setting expectations), and when the more advanced strategies (e.g., leadership, transfer) are implemented in purposeful ways, improvements in life skills learning are possible. Given that the purpose of Olivia's training was to integrate those strategies which were relatively low at baseline, this was seen as a success. Particularly, it is notable that Olivia's ratings on TARE 2.0 items “role in assessment” and “transfer” improved from non-existent at baseline to consistent implementation. Although the final ratings were relatively low, this reflects the frequency with which these strategies were observed rather than the quality of implementation. Escartí et al. (2015) expect that the more advanced teaching strategies do not happen in every observation interval or even in every class period. To this point, the claim can be made that PE teachers do not need to make sweeping changes to practices in order to see positive and short-term results (Pascual et al., 2011; Gray et al., 2019). Rather, targeted attention to higher level responsibility-based strategies can result in more positive student experiences in PE (Coulson et al., 2012).

Another noteworthy finding is the concept that teacher practices in the TARE strongly link to student experiences in PE. According to Escartí et al. (2015), significant correlations have been observed between the teacher's use of responsibility-based teaching strategies and students' display of responsible behaviors. Specifically, patterns between teachers providing leadership opportunities and students taking leadership, and teachers giving choices and voices and students expressing voice were noted in the current study. These patterns highlight the importance of teachers building relationships and interacting with students as a precursor to students developing affective outcomes (Hellison, 2011).

The findings from the YES 2.0 suggested that short-term changes to youth experiences in the PE program were observed for the students in the intervention group over time. Participants reported having experiences related to problem solving, emotional regulation, effort, goal setting, identity experiences, time management, and promoting social norms. The findings related to problem solving and effort are supported by TARE data which demonstrated students in the intervention group consistently participated, were engaged, and cooperated with peers during activities throughout the intervention. The finding that students' experiences with emotional regulation increased over time is highlighted in other research where an improved capacity for self-control and increases in self-regulation behaviors were reported by participants in a TPSR program (Escartí et al., 2010). In general, these findings align with the concept that intentionally designed sport experiences can foster PYD outcomes and life skill development such as self-control and promote teamwork and effort (Gould and Carson, 2008; Hellison, 2011; Jacobs and Wright, 2018).

One finding that was unexpected and warrants exploration is how the YES 2.0 instrument did not capture changes in student leadership and responsibility experiences. During feedback sessions throughout the intervention, Olivia consistently expressed concern about putting youth in charge of others during activities because she did not perceive they were ready to handle the level of responsibility. This is a commonly held belief in TPSR and other PE based instructional models where teachers are hesitant to shift control and power to students because it conflicts with traditional authoritarian pedagogy and both students and teachers must learn to practice non-traditional roles (Sinelnikov, 2009). It may be that due to the short implementation period of the intervention, the teacher was not prepared to make this change without further training. Despite these challenges, Hemphill et al. (2015) offer that many teachers perceive TPSR will positively impact their students, and with proper training, would be committed to consistently integrating these strategies into their regular practice.

### Limitations and Future Directions

This study extends TPSR literature through exploring student experiences of life skill learning through a PE-based responsibility intervention. Previous literature has not yet examined the link between teacher practice and student experiences with life skills in PE. Along with this contribution, the limitations of the study should be acknowledged. First, the current study did not assess Olivia's training in a systematic way. Without this information, it is difficult to presume the level of understanding the intervention teacher had of TPSR content. It is also possible that some of the differences in student perceptions are attributable to teacher differences (e.g., motivation, experience) rather than differences attributable to the intervention.

Another limitation to the integrity of the research design relates to the increase of some responsibility-based teaching strategies in the control condition. Ben's improvement on some of these fundamental strategies (e.g., modeling respect, setting expectations, fostering social interaction) may have been due in part to the Hawthorne effect, a common phenomenon in observational research whereby the individual being observed makes slight changes in their behaviors in response to being observed (McCambridge et al., 2014). As for Olivia's ratings in these areas, they were consistently higher at pre and post-test and likely encountered a ceiling effect (Salkind, 2010). While the TARE 2.0 observations provided sufficient data for a nuanced description of the teachers' practice before and after the training intervention, ongoing data collection in both conditions was lacking. In future studies, ongoing observation or the use of a fidelity checklist would be advisable (Hastie and Casey, 2014). As new instruments such as the Observational System of Teaching Oriented Responsibility (OSTOR; Camerino et al., 2019) are developed specifically to monitor TPSR implementation, they should also be considered for inclusion in future studies.

The more empowerment-based teaching strategies (i.e., Leadership, Giving Choices and Voices, Role in Assessment, and Transfer) are seen less often in common practice and are therefore the stronger indicators of difference between conditions and over time (Wright and Irwin, 2018; Wright et al., 2021). Ben did show some increase on Leadership and Giving Choices and Voices, but these were with effect sizes below 1.0. The changes on these two strategies may have also been related to the Hawthorne effect or possibly to treatment contamination, i.e., some elements of the treatment condition may have unintentionally been introduced in the control condition (Rhoads, 2011). Because both teachers worked closely at the same school, it is possible that Ben may have witnessed and either consciously or unconsciously mimicked some of the new practices Olivia was implementing because they seemed to be effective practice. Regarding these higher-level responsibility strategies, Olivia increased significantly on all four. This included achieving higher effect sizes than Ben on Leadership and Choices and Voices as well as introducing two strategies that Ben never employed, i.e., Role in Assessment and Transfer. In sum, while changes in Ben's practice presented a limitation, data still indicated there was a clear difference in the implementation of responsibility-based teaching. Students in Olivia's classes were exposed to higher levels of responsibility-based instruction overall and a marked change in the type and strength of higher-level responsibility-based teaching strategies over time.

It is also important to acknowledge the limits of the YES 2.0 survey in terms of the current study. First, the responses to the YES 2.0 are based on youth self-report, which risks potential for social desirability and inaccurate recall among participants (Schwarz, 1999). Additionally, this survey assesses students' perceptions on experiencing life skill content during PE, not necessarily their level of skill attainment or enactment. Further, the research design, which included 12 subscales from the YES 2.0 measured at pre- and post-intervention, precluded the ability to use more sophisticated designs, such as MANCOVA or hierarchical linear modeling. While future studies should examine the extent to which students report learning and making use of these life skills in other contexts (Gordon, 2010; Jacobs and Wright, 2018), the current study presents a step forward in the literature. Specifically, prior research has demonstrated that adolescent development is marked by students reflecting and creating meanings behind the activities in which they participate and perceptions and beliefs have been found to influence actions (Wright and Burton, 2008; Jacobs and Wright, 2021).

### Conclusions

Despite the noted limitations, this study provides support for the idea that PE teachers can integrate PYD practices, such as TPSR, into their regular teaching and see increases in students' perceptions of life skill development. This can add coherence and intentionality to the way teachers promote affective development, personal and social skills, as well as SEL in PE (Opstoel et al., 2020; Teraoka et al., 2020; Dyson et al., 2021). In order to strengthen the rationale for using PYD practices such as TPSR within PE, future studies should examine how these life skills can be applied beyond PE. One such methodology is The Transfer of Responsibility Questionnaire (Wright et al., 2019) which captures whether youth report transferring responsibility-based life skills learned in PE to other contexts. This could be an ideal way to connect the impact of a responsibility-based pedagogy on PYD outcomes and life skill application outside of PE, which has been a longtime challenge in the literature (Jacobs and Wright, 2018). Future scholars are similarly invited to examine the ways in which student demographic variables, such as age, gender, and race/ethnicity, as well as contextual factors, such as whether or schools are positioned in urban, suburban, or rural environments, influences perceptions of life skills learning in PE. These variables were not examined in the current investigation, but could have implications for student learning. Based on these results, practitioners are encouraged to look to the TPSR model as one resource and set of best practices for addressing PYD in the curriculum.

## Data Availability Statement

The raw data supporting the conclusions of this article will be made available by the authors, without undue reservation.

## Ethics Statement

The studies involving human participants were reviewed and approved by Institutional Review Board at Northern Illinois University. Written informed consent to participate in this study was provided by the participants' legal guardian/next of kin.

## Author Contributions

JJ, PW, and KR jointly designed the study. JJ trained the interventionist, collected, and processed the data. JJ and KR collaborated on data analysis. All authors contributed to writing the current manuscript.

## Conflict of Interest

The authors declare that the research was conducted in the absence of any commercial or financial relationships that could be construed as a potential conflict of interest.

## Publisher's Note

All claims expressed in this article are solely those of the authors and do not necessarily represent those of their affiliated organizations, or those of the publisher, the editors and the reviewers. Any product that may be evaluated in this article, or claim that may be made by its manufacturer, is not guaranteed or endorsed by the publisher.
